# Disparities in COVID-19 Vaccination Status, Intent, and Perceived Access for Noninstitutionalized Adults, by Disability Status — National Immunization Survey Adult COVID Module, United States, May 30–June 26, 2021

**DOI:** 10.15585/mmwr.mm7039a2

**Published:** 2021-10-01

**Authors:** A. Blythe Ryerson, Catherine E. Rice, Mei-Chuan Hung, Suchita A. Patel, Julie D. Weeks, Jennifer L. Kriss, Georgina Peacock, Peng-Jun Lu, Amimah F. Asif, Hannah L. Jackson, James A. Singleton

**Affiliations:** ^1^Division of Human Development and Disability, National Center on Birth Defects and Developmental Disabilities, CDC; ^2^CDC COVID-19 Response Team; ^3^Immunization Services Division, National Center for Immunization and Respiratory Diseases, CDC; ^4^Leidos Inc, Atlanta, Georgia; ^5^Division of Analysis and Epidemiology, National Center for Health Statistics, CDC.

Estimates from the 2019 American Community Survey (ACS) indicated that 15.2% of adults aged ≥18 years had at least one reported functional disability (*1*). Persons with disabilities are more likely than are those without disabilities to have chronic health conditions (*2*) and also face barriers to accessing health care (*3*). These and other health and social inequities have placed persons with disabilities at increased risk for COVID-19–related illness and death, yet they face unique barriers to receipt of vaccination (*4*,*5*). Although CDC encourages that considerations be made when expanding vaccine access to persons with disabilities,* few public health surveillance systems measure disability status. To describe COVID-19 vaccination status and intent, as well as perceived vaccine access among adults by disability status, data from the National Immunization Survey Adult COVID Module (NIS-ACM) were analyzed. Adults with a disability were less likely than were those without a disability to report having received ≥1 dose of COVID-19 vaccine (age-adjusted prevalence ratio [aPR] = 0.88; 95% confidence interval [CI] = 0.84–0.93) but more likely to report they would definitely get vaccinated (aPR = 1.86; 95% CI = 1.43–2.42). Among unvaccinated adults, those with a disability were more likely to report higher endorsement of vaccine as protection (aPR = 1.29; 95% CI = 1.16–1.44), yet more likely to report it would be or was difficult to get vaccinated than did adults without a disability (aPR = 2.69; 95% CI = 2.16–3.34). Reducing barriers to vaccine scheduling and making vaccination sites more accessible might improve vaccination rates among persons with disabilities.

Data from noninstitutionalized adults aged ≥18 years were collected in the NIS-ACM by telephone interview during May 30–June 26, 2021 using a random-digit–dialed sample of cellular telephone numbers, stratified by locality.^†^ Although the current U.S. Department of Health and Human Services (HHS) minimum standard for measuring disability in surveys relies on six questions (*6*), during the COVID-19 emergency response, data collection opportunities were limited. To assess COVID-19 vaccination status for this demographic group, CDC added a single question to the NIS-ACM: “Do you have serious difficulty seeing, hearing, walking, remembering, making decisions, or communicating?” Respondents who answered “yes” were considered to have a disability, and those who answered “no” were categorized as having no disability. Among all respondents (56,749; 18.9% final response rate), 5,361 (9.4%) reported having a disability, and 51,253 (90.3%) reported no disability. Disability status was missing for 135 (0.2%) respondents, and these respondents were excluded from all analyses. Respondents were also asked a series of questions on perceived COVID-19 risk, current COVID-19 vaccination status, and attitudes and perceived barriers to getting vaccinated.^§^

All percentages were weighted to represent the noninstitutionalized U.S. adult population. Survey weights were calibrated to state-level vaccine administration data reported to CDC as of June 15, 2021.^¶^ T-tests were performed to detect differences in percentages between groups. Unadjusted and age-adjusted vaccination prevalence ratios (PRs) comparing percentages of adults with a disability with those without a disability were calculated using logistic regression and predictive marginals. T-tests and PRs were considered statistically significant if p-values were <0.05. All analyses were performed using SAS (version 9.4; SAS Institute) and SUDAAN (version 11.0.3; Research Triangle Institute). This activity was reviewed by CDC and was conducted consistent with applicable federal law and CDC policy.**

Among all respondents, 9.4% reported having a disability. In age-adjusted analyses, adults with a disability were less likely than were those without a disability to report having received ≥1 dose of a COVID-19 vaccine (aPR = 0.88; 95% CI = 0.84–0.93) (Table) but more likely to report they would definitely get vaccinated (aPR = 1.86; 95% CI = 1.43–2.42) (Supplementary Table 1, https://stacks.cdc.gov/view/cdc/109902). Among unvaccinated adults, those with a disability were more likely than those without a disability to report they were very or moderately concerned about getting COVID-19 (aPR = 1.61; 95% CI = 1.37–1.89), thought the vaccine is very or somewhat important for protection (aPR = 1.29; 95% CI = 1.16–1.44), reported many or almost all friends and family members as vaccinated (aPR = 1.19; 95% CI = 1.03–1.38), and had a health care provider recommend the vaccine (aPR = 1.27; 95% CI = 1.08–1.51) (Figure 1) (Supplementary Table 2, https://stacks.cdc.gov/view/cdc/109903).

**TABLE:** COVID-19 vaccination status* of adults aged ≥18 years, by respondent characteristic and disability status^†^ — National Immunization Survey Adult COVID Module, United States, May 30–June 26, 2021

Respondent group/Characteristic	With a disability^†^		Without a disability		Prevalence ratio^§^ (95% CI)	
	No.	%^¶^ Vaccinated* (95% CI)	No.	%^¶^ Vaccinated* (95% CI)	Unadjusted	Age-adjusted
**Total**	**5,345**	**66.7 (63.9–69.3)**	**51,106**	**64.5 (63.5–65.4)**	**1.03 (0.99–1.08)**	0.88 (0.84–0.93)**
**Age group, yrs**						
18–24 (Ref)	198	33.5 (23.8–44.7)	5,015	46.4 (43.9–49.0)	0.72 (0.52–0.99)**	NA
25–29	162	35.5 (22.9–50.6)	4,203	49.8 (46.7–52.9)	0.71 (0.48–1.07)	NA
30–39	372	48.8 (40.2–57.5)^††^	8,817	52.9 (50.8–55.0)^††^	0.92 (0.77–1.11)	NA
40–49	559	54.4 (46.2–62.3)^††^	8,050	60.8 (58.4–63.1)^††^	0.89 (0.77–1.04)	NA
50–64	1,783	62.8 (57.6–67.7)^††^	14,246	71.9 (70.1–73.6)^††^	0.87 (0.80–0.95)**	NA
65–74	1,260	82.7 (77.4–87.0)^††^	7,069	88.6 (86.6–90.4)^††^	0.93 (0.88–0.99)**	NA
≥75	953	88.2 (83.7–91.6)^††^	2,827	90.0 (87.5–92.1)^††^	0.98 (0.93–1.03)	NA
**Sex**						
Male (Ref)	2,542	66.4 (62.2–70.5)	25,297	61.9 (60.6–63.2)	1.07 (1.00–1.15)**	0.91 (0.85–0.99)**
Female	2,747	67.3 (63.6–70.7)	25,457	67.0 (65.7–68.3)^††^	1.00 (0.95–1.06)	0.86 (0.80–0.92)**
**Race/Ethnicity^§§^**						
White (Ref)	2,993	69.0 (65.4–72.4)	30,871	66.6 (65.5–67.7)	1.04 (0.98–1.09)	0.88 (0.82–0.94)**
Hispanic	822	67.2 (59.8–73.7)	6,613	61.8 (59.2–64.3)^††^	1.09 (0.97–1.22)	0.94 (0.84–1.05)
Black	726	60.1 (52.8–67.1)^††^	5,748	56.3 (53.6–58.9)^††^	1.07 (0.94–1.22)	0.84 (0.73–0.97)**
AI/AN	105	38.2 (23.6–55.2)^††^	538	39.2 (31.8–47.1)^††^	0.97 (0.81–1.56)	0.85 (0.56–1.30)
Asian	127	74.7 (46.5–90.9)	3,015	85.5 (81.5–88.8)^††^	0.87 (0.64–1.19)	0.86 (0.67–1.12)
NHPI	113	71.1 (27.8–94.0)	974	59.1 (47.0–70.2)	1.20 (0.68–2.13)	0.78 (0.32–1.91)
Multiple race/Other	294	55.6 (43.0–67.6)^††^	1,797	49.2 (43.9–54.5)^††^	1.13 (0.88–1.45)	0.85 (0.62–1.18)
**Urbanicity^¶¶^**						
MSA, principal city (Ref)	1,387	68.7 (62.8–74.0)	14,609	68.0 (66.2–69.7)	1.01 (0.93–1.10)	0.88 (0.79–0.97)**
MSA, nonprincipal city	2,697	67.4 (63.7–70.9)	26,796	65.1 (63.9–66.3)^††^	1.04 (0.98–1.10)	0.89 (0.84–0.95)**
Non-MSA	1,261	61.4 (55.4–67.2)	9,701	54.4 (52.0–56.7)^††^	1.13 (1.02–1.26)	0.88 (0.77–1.01)
SVI of county of residence***						
Low (Ref)	1,225	68.0 (62.3–73.2)	14,066	69.9 (68.2–71.6)	0.97 (0.89–1.06)	0.84 (0.76–0.93)**
Moderate	1,687	68.6 (63.6–73.2)	17,064	65.1 (63.5–66.6)^††^	1.05 (0.98–1.14)	0.90 (0.83–0.99)**
High	1,520	64.8 (59.8–69.4)	11,864	60.4 (58.6–62.2)^††^	1.07 (0.99–1.16)	0.89 (0.82–0.98)**
**Poverty status and household income^†††^**						
Above poverty, ≥$75k (Ref)	798	78.0 (70.3–84.1)	19,539	72.5 (71.0–73.9)	1.08 (0.98–1.17)	0.97 (0.87–1.07)
Above poverty, <$75k	1,911	68.9 (64.5–73.1)^††^	15,528	61.1 (59.4–62.7)^††^	1.13 (1.05–1.21)**	0.94 (0.86–1.03)
Below poverty	1,137	55.5 (49.4–61.5)^††^	4,410	48.6 (45.6–51.7)^††^	1.14 (1.01–1.30)**	0.95 (0.83–1.07)
Unknown income	1,499	66.6 (61.3–71.4)^††^	11,629	64.3 (62.4–66.2)^††^	1.03 (0.95–1.12)	0.89 (0.81–0.98)**
**Education level**						
College graduate (Ref)	1,338	80.2 (74.6–84.9)	23,844	79.2 (78.0–80.5)	1.01 (0.95–1.08)	0.89 (0.81–0.99)**
Some college	1,652	66.0 (61.2–70.5)^††^	13,590	62.2 (60.5–63.9)^††^	1.06 (0.98–1.14)	0.94 (0.87–1.03)
High school graduate or less	2,175	62.6 (58.4–66.6)^††^	12,231	53.8 (52.1–55.5)^††^	1.16 (1.08–1.25)**	0.93 (0.86–1.01)
**Health insurance**						
Insured (Ref)	4,803	69.4 (66.5–72.1)	45,472	67.6 (66.6–68.6)	1.03 (0.98–1.07)	0.88 (0.83–0.93)**
Not insured	363	40.2 (31.7–49.3)^††^	4,205	42.1 (39.2–45.1)^††^	0.95 (0.76–1.20)	0.87 (0.72–1.05)
**Mental health**						
Excellent, very good, or good (Ref)	3,866	70.7 (67.4–73.7)	46,379	64.8 (63.9–65.8)	1.09 (1.04–1.11)**	0.92 (0.86–0.98)**
Fair or poor	1,398	56.5 (51.2–61.7)^††^	4,405	61.5 (58.2–64.6)^††^	0.92 (0.83–1.02)	0.79 (0.72–0.88)**
**Comorbidities^§§§^**						
No (Ref)	2,087	62.2 (57.8–66.4)	37,054	60.2 (59.2–61.3)	1.03 (0.96–1.11)	0.89 (0.83–0.96)**
Yes	3,120	71.0 (67.5–74.3)^††^	13,577	76.6 (74.9–78.2)^††^	0.93 (0.88–0.98)**	0.82 (0.77–0.88)**
**Ever had COVID-19**						
No (Ref)	4,496	68.6 (65.6–71.5)	43,223	68.1 (67.1–69.1)	1.01 (0.96–1.06)	0.86 (0.81–0.91)**
Yes	776	59.1 (52.3–65.5)^††^	7,234	49.1 (46.9–51.4)^††^	1.20 (1.06–1.96)**	1.05 (0.93–1.18)
**Received any vaccine that was not a COVID-19 vaccine in the past 2 years**						
Yes (Ref)	3,224	81.1 (78.0–83.9)	29,282	80.7 (79.6–81.7)	1.01 (0.97–1.05)	0.90 (0.85–0.95)**
No	2,078	48.1 (43.8–52.4)^††^	21,534	47.5 (46.2–48.8)^††^	1.01 (0.92–1.11)	0.86 (0.78–0.95)**

**Abbreviations:** AI/AN = American Indian or Alaska Native; CI = confidence interval; MSA = metropolitan statistical area; NA = not applicable; NHPI = Native Hawaiian or Other Pacific Islander; Ref = referent group; SVI = social vulnerability index.

* At least 1 dose of any of the approved COVID-19 vaccines (Pfizer-BioNTech, Moderna, or Janssen [Johnson & Johnson]).

^†^ Disability was defined as an affirmative response to the following survey question: “Do you have serious difficulty seeing, hearing, walking, remembering, making decisions, or communicating?”

^§^ Prevalence ratio comparing vaccination rates among persons with a disability with rates among persons without a disability.

^¶^ Weighted percentage. Respondents missing either vaccination or disability status were excluded (298).

** p<0.05 for prevalence ratio.

^††^ p<0.05 by t-test for comparisons of proportions with the indicated reference level.

^§§^ White, Black, AI/AN, Asian, NHPI, and multiple-race persons were non-Hispanic; Hispanic persons could be of any race.

^¶¶^ Urbanicity derived based on the centroid of the zip code of residence.

*** CDC and Agency for Toxic Substances and Disease Registry Social Vulnerability Index use 15 U.S. Census variables to help officials identify communities that might need support before, during, or after disasters. https://www.atsdr.cdc.gov/placeandhealth/svi/index.html

^†††^ Household income is derived from the number of persons reported in the household, the reported household income, and the 2020 U.S. Census poverty thresholds.

^§§§^ Based on response to the question, “Do you have a health condition that may put you at higher risk for COVID-19?”

**FIGURE 1:**
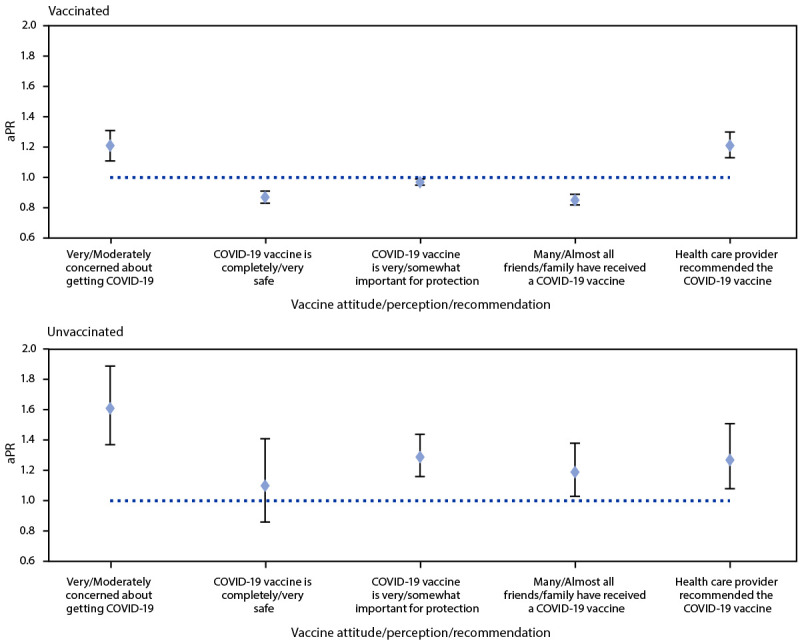
Age-adjusted prevalence ratios* of COVID-19 vaccine attitudes, perceptions, and recommendations^†^ among adults aged ≥18 years with a disability^§^ compared with adults without a disability, by COVID-19 vaccination status^¶^ — National Immunization Survey Adult COVID Module, United States, May 30–June 26, 2021 **Abbreviation**: aPR = age-adjusted prevalence ratio. * 95% confidence intervals indicated with error bars. ^†^ Prevalence ratio p<0.05 for all groups except “unvaccinated: thinks a COVID-19 vaccine is completely/very safe.” ^§^ Disability was defined as an affirmative response to the following survey item: “Do you have serious difficulty seeing, hearing, walking, remembering, making decisions, or communicating?” ^¶^ Respondents were considered vaccinated if they reported having received at least 1 dose of any of the approved COVID-19 vaccines (Pfizer-BioNTech, Moderna, or Janssen [Johnson & Johnson]).

Overall, adults with a disability were more likely than were those without a disability to report that it would be or was somewhat or very difficult to get vaccinated (aPR = 1.19; 95% CI = 1.05–1.36), and this observation was more pronounced among the unvaccinated (aPR = 2.69; 95% CI = 2.16–3.34) (Figure 2). Among unvaccinated adults, those with a disability were more likely than were those without a disability to report having the following difficulties associated with getting the vaccine: getting an appointment online (aPR = 2.14; 95% CI = 1.48–3.10), not knowing where to get vaccinated (aPR = 1.95; 95% CI = 1.36–2.79), getting to vaccination sites (aPR = 3.43; 95% CI = 2.53–4.67), and vaccination sites not being open at convenient times (aPR = 1.69; 95% CI = 1.23–2.33).

**FIGURE 2:**
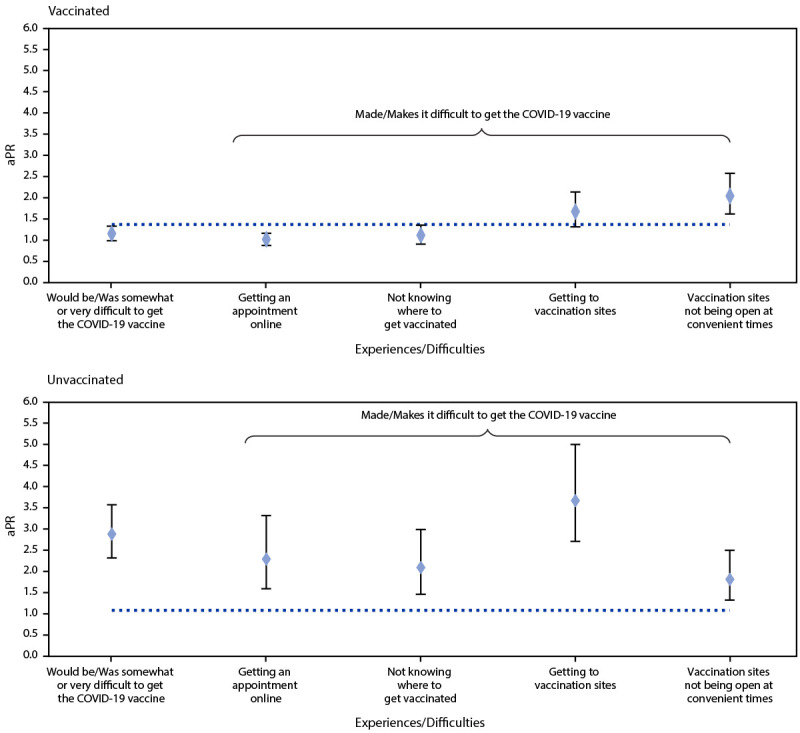
Age-adjusted prevalence ratios* of experiences and difficulties with getting the COVID-19 vaccine^†^ among adults aged ≥18 years with a disability^§^ compared with adults without a disability, by COVID-19 vaccination status^¶^ — National Immunization Survey Adult COVID Module, United States, May 30–June 26, 2021 **Abbreviation**: aPR = age-adjusted prevalence ratio. * 95% confidence intervals indicated with error bars. ^†^ Prevalence ratio p<0.05 for all groups except “vaccinated: getting to vaccination sites.” ^§^ Disability was defined as an affirmative response to the following survey item: “Do you have serious difficulty seeing, hearing, walking, remembering, making decisions, or communicating?” ^¶^ Respondents were considered vaccinated if they reported having received at least 1 dose of any of the approved COVID-19 vaccines (Pfizer-BioNTech, Moderna, or Janssen [Johnson & Johnson]).

## Discussion

COVID-19 vaccination coverage was lower among U.S. adults with a disability than among those without a disability, even though adults with a disability reported less hesitancy to getting vaccinated. Unvaccinated adults with disabilities were more likely than were those without a disability to report thinking that the vaccine is important protection, indicating that there might be potential for increasing vaccination coverage in this group. However, adults with a disability anticipated or experienced more difficulty obtaining a COVID-19 vaccination than did those without a disability. Reducing barriers to scheduling and making vaccination sites more accessible might improve vaccination rates among persons with disabilities (*7*).

Much work has been done to adapt COVID-19 health messages into more accessible formats^††^; however, more effort is necessary to increase health equity for persons with disabilities. A recent exploratory analysis of official state and territorial COVID-19 vaccination registration websites found substantial variability and suboptimal compliance with basic accessibility recommendations (*8*). Information is available for developers of online health information resources and scheduling systems to make web content more accessible.^§§^ Further, online scheduling systems can provide call lines for persons who need assistance making an appointment or requesting assistance getting to a vaccination site. CDC recently provided funding to the Administration for Community Living (ACL) to create a national Disability Information and Access Line (DIAL) to assist persons with disabilities obtain a COVID-19 vaccination.^¶¶^

Even if vaccination locations are identified and appointments are secured, vaccination sites might vary in their accessibility options. All vaccination sites are required to be compliant with the Americans with Disabilities Act; however, regulations do not require that sites have American Sign Language (ASL) interpreters or providers trained to work with persons with intellectual or other developmental disabilities (*9*). Transportation to a vaccination site might be particularly challenging for persons with a disability who depend on another person to take them or who need accessible vehicles or public transportation. To help overcome some of these challenges, CDC recently provided funding to ACL to provide grants to aging and disability networks in every U.S. state and territory to expand access to COVID-19 vaccination among older adults and persons with disabilities.*** These grants aid with scheduling vaccination appointments, providing direct support services needed to attend appointments, providing transportation to vaccination sites, and connecting persons who cannot leave their homes independently to in-home vaccination options.

The findings in this report are subject to at least four limitations. First, the low response rate and exclusion of persons living in institutionalized settings and phoneless or landline-only households introduces the possibility for selection bias. Estimates of COVID-19 vaccination coverage might differ from vaccine administration and other data reported at https://covid.cdc.gov/covid-data-tracker/#vaccinations.^†††^ Second, the question assessing disability status is new and has not been validated or cognitively tested. Approximately 9% of respondents in the NIS-ACM reported a disability based on the new question, which is lower than the 15% 2019 ACS estimate for adults using the HHS minimum standard six-question set; this variation is likely attributable to multiple factors, including differences in eligibility criteria, survey methods, and questionnaire language. However, even with differing disability prevalence estimates on various national surveys, observed health disparities remain consistent among persons with disabilities (*10*). Third, attempting to measure this heterogenous demographic group with a single question limits the ability to consider functional type or severity of different disabilities and might obscure differences in access and perceptions of some subgroups. Finally, statistical power is lower to detect differences for persons with a disability than for persons without a disability because of smaller sample sizes.

Public health efforts that make COVID-19 vaccination information, scheduling, and sites more easily accessible for persons with disabilities might help to address health inequities and increase vaccination demand and coverage (*7*). These include making health messages and vaccination information available in ASL, braille, and easy-to-read formats, making all vaccination sites more accessible to persons of all ability types, including persons with intellectual disabilities and sensory disabilities, and making COVID-19 vaccination available to those who are unable to leave their homes easily or independently. These efforts would be relevant to the reduction of health disparities related to disability beyond the COVID-19 pandemic. Further, regular collection of disability status as a demographic variable in public health surveillance systems can facilitate ongoing monitoring of health disparities among persons with disabilities and help guide understanding of the contextual factors underlying health inequities.

**Summary**

**What is already known about this topic?**

Persons with disabilities are at increased risk for COVID-19–related illness and death.

**What is added by this report?**

Analysis of the National Immunization Survey Adult COVID Module found that, compared with adults without a disability, those with a disability had a lower likelihood of having received COVID-19 vaccination, despite being less likely to report hesitancy about getting vaccinated. Adults with a disability reported more difficulties obtaining a COVID-19 vaccine than did persons without a disability.

**What are the implications for public health practice?**

Reducing barriers to scheduling and making vaccination sites more accessible might improve vaccination coverage among persons with disabilities.

## Footnotes

* COVID-19 Vaccine Recommendations and Guidelines of the Advisory Committee on Immunization Practices, CDC. https://www.cdc.gov/vaccines/hcp/acip-recs/vacc-specific/covid-19.html

† The NIS-ACM cellular telephone sample was stratified by state, the District of Columbia, five local jurisdictions (Bexar County, Texas; Chicago, Illinois; Houston, Texas; New York City, New York; and Philadelphia County, Pennsylvania), and Guam, Puerto Rico, and the U.S. Virgin Islands.

§
https://www.cdc.gov/vaccines/imz-managers/nis/downloads/NIS-ACM-Questionnaire-Q2-2021_508.pdf

¶ Additional information about the NIS-ACM is available at https://www.cdc.gov/vaccines/imz-managers/nis/about.html#current-surveys.

** 45 C.F.R. part 46; 21 C.F.R. part 56; 42 U.S.C. Sect. 241(d); 5 U.S.C. Sect. 552a; 44 U.S.C. Sect. 3501 et seq.

†† Accessible COVID-19 vaccine messages adapted from CDC’s full guidance are available at https://cidi.gatech.edu/covid.

§§ The Web Accessibility Initiative provides information on making web content accessible. https://www.w3.org/WAI/standards-guidelines/wcag/

¶¶ Persons with a disability seeking assistance in getting a COVID-19 vaccine can call 888-677-1199, Monday–Friday from 9:00 a.m. to 8:00 p.m. EST or can email DIAL@n4a.org.

***
https://www.hhs.gov/about/news/2021/03/29/hhs-to-expand-access-to-covid-19-vaccines-for-older-adults-and-people-with-disabilities.html

††† Estimates of COVID-19 vaccination and intent are also available at https://covid.cdc.gov/covid-data-tracker/#vaccinations-disability-status using data from the Census Bureau Household Pulse Survey, and might differ from NIS-ACM estimates. Both data sources indicate lower COVID-19 vaccination coverage among persons with disabilities compared with those without a disability.
